# Antimicrobial susceptibility and antibiotic resistance gene transfer analysis of foodborne, clinical, and environmental *Listeria* spp. isolates including *Listeria monocytogenes*

**DOI:** 10.1002/mbo3.155

**Published:** 2014-01-02

**Authors:** David Bertsch, Mirjam Muelli, Monika Weller, Anaïs Uruty, Christophe Lacroix, Leo Meile

**Affiliations:** Laboratory of Food Biotechnology, Institute of Food, Nutrition and Health, ETH ZurichZurich, Switzerland

**Keywords:** Antibiotics, environmental, food, *Listeria*, resistance.

## Abstract

The aims of this study were to assess antibiotic resistance pheno-and genotypes in foodborne, clinical, and environmental *Listeria* isolates, as well as to elucidate the horizontal gene transfer potential of detected resistance genes. A small fraction of in total 524 *Listeria* spp. isolates (3.1%) displayed acquired antibiotic resistance mainly to tetracycline (*n *=* *11), but also to clindamycin (*n *=* *4) and trimethoprim (*n* = 3), which was genotypically confirmed. In two cases, a tetracycline resistance phenotype was observed together with a trimethoprim resistance phenotype, namely in a clinical *L. monocytogenes* strain and in a foodborne *L. innocua* isolate. Depending on the applied guidelines, a differing number of isolates (*n *=* *2 or *n *=* *20) showed values for ampicillin that are on the edge between intermediate susceptibility and resistance. Transferability of the antibiotic resistance genes from the *Listeria* donors, elucidated in vitro by filter matings, was demonstrated for genes located on transposons of the Tn*916* family and for an unknown clindamycin resistance determinant. Transfer rates of up to 10^−5^ transconjugants per donor were obtained with a *L. monocytogenes* recipient and up to 10^−7^ with an *Enterococcus faecalis* recipient, respectively. Although the prevalence of acquired antibiotic resistance in *Listeria* isolates from this study was rather low, the transferability of these resistances enables further spread in the future. This endorses the importance of surveillance of *L. monocytogenes* and other *Listeria* spp. in terms of antibiotic susceptibility.

## Introduction

*Listeria monocytogenes* is an emerging foodborne pathogen causing listeriosis and accounts after *Salmonella* for the second-most food-related deaths in the United States (Scallan et al. 2011). In the European Union, listeriosis even led by far to the most food-related deaths (*n *=* *134) caused by bacteria in 2011, much more than salmonellosis (*n *=* *56) (EFSA and ECDC 2013). A typical treatment of this invasive disease is the application of penicillin or ampicillin together with gentamicin (Swaminathan and Gerner-Smidt 2007); however, adaptations can be advisable for certain patients and clinical manifestations (recently reviewed by Allerberger and Wagner 2010). Regardless of an early antibiotic therapy, which is crucial for physical recovery of the patients (Mayrhofer et al. 2004), mortality rates are very high, ˜20% to 30% (Watson 2009). Next to *L. monocytogenes*, also the knowledge about transferable resistance genes present in other *Listeria* species is important, as *L. innocua* was recognized as a reservoir of antibiotic resistance (AB^R^) for *L. monocytogenes* (Bertrand et al. 2005). *Listeria* strains are frequently detected in food products due to their ubiquitary occurrence in the environment. In addition, growth and survival of these psychrotrophic bacteria are favored particularly due to the increasing demand for minimally processed foods and absence of preservatives. It was estimated that 99% of listeriosis cases occur due to foodborne transmission of the bacteria (Scallan et al. 2011).

*Listeria* spp. are often exposed to low levels of antibiotics, as these agents are used in large amounts both in human and animal medicine (Aarestrup 2012). This can lead to an increased selective pressure which favors emergence of AB^R^ due to mutations or acquisition of mobile genetic elements. Thus, it was not surprising, when the first acquired AB^R^ in *L. monocytogenes* was reported in 1990 (Poyart-Salmeron et al. 1990). Further multidrug-resistant *Listeria* strains due to acquisition of plasmids were sporadically detected (Quentin et al. 1990; Hadorn et al. 1993; Tsakris et al. 1997). A way to limit the spread of antimicrobial resistances is to reduce the application of these substances. This consideration led to the ban of antibiotics as feed additives in Switzerland already in 1999 (Arnold et al. 2004) and from January 2006 onward also in the European Union (Castanon 2007). In contrast, antimicrobial compounds are still administered as growth promoters, for example, in the United States, without control by veterinarians (Jones and Ricke 2003). An announcement of the Food and Drug Administration from April 2012 suggests stopping application of antibiotics for growth promotion on a voluntary basis (http://www.fda.gov).

There are relatively few epidemiological studies and thus only limited information on AB^R^ prevalence and spread concerning *Listeria* spp. and many studies focused on local situations and special origins of *Listeria* isolates. Therefore, the aim of this study was the assessment of resistance pheno-and genotypes in foodborne, clinical, and environmental *Listeria* isolates from the different regions of Switzerland, as well as elucidating the horizontal gene transfer potential of detected AB^R^ genes.

## Material and Methods

### Bacterial strains

*Listeria* strains were isolated from food for this study by application of ONE Broth-Listeria and *Brilliance*™ Listeria Agar (ready to use plates; both Oxoid, Pratteln, Switzerland). Other strains were obtained from own culture collections at ETH Zurich or provided by the food industry, hospitals, and veterinary clinics in Switzerland. In total 524 isolates were studied, 24 originated from poultry, 132 from meat products, 46 from dairy products, 18 from fish/seafood, 36 from production (swaps), five from processed food, eight from plant material, 100 from water, and 155 from humans. The *Listeria* strains of human clinical origin were provided anonymized as pure cultures from the culture collection of the Lausanne University Hospital (CHUV, Lausanne, Switzerland). The isolates were from 2006 (*n* = 33), 2007 (*n* = 54), 2008 (*n *=* *151), 2009 (*n* = 142), 2010 (*n* = 68), 2011 (*n *=* *72), and 2012 (*n* = 4) and comprised the species *L. monocytogenes* (*n* = 383), *L. innocua* (*n *=* *50), *L. ivanovii* subsp. *ivanovii* (*n *=* *5), *L. ivanovii* subsp. *londoniensis* (*n *=* *20), *L. welshimeri* (*n *=* *6), and *L. seeligeri* (*n *=* *56). Four isolates were assigned to *L. fleischmannii* sp. nov. (Bertsch et al. 2013a). All strains were cultured on brain heart infusion (BHI) agar medium (Biolife Italiana S.r.l., Milano, Italy) at 37°C and stored at −80°C in BHI medium containing 33% glycerol.

### DNA isolation and PCR assays

Chromosomal and extrachromosomal DNA from single bacterial colonies was isolated and purified as described before (Bertsch et al. 2013b). Sequencing analyses were performed by Microsynth AG (Balgach, Switzerland) or GATC Biotech AG (Konstanz, Germany). PCR assays with primers (purchased from Microsynth AG) used in this study are listed in Table S1. For templates up to 3 kb, PCR Master Mix (2X) with *Taq* polymerase (Fermentas, Le-Mont-Sur-Lausanne, Switzerland) was applied. Longer targets were amplified with either Phusion™ High-Fidelity DNA Polymerase (New England BioLabs, Allschwil, Switzerland) or GoTaq® Long PCR Master Mix (Promega, Duebendorf, Switzerland). *L. monocytogenes* isolates were further analyzed by application of multiplex PCR assays for rapid discrimination of the major serovars 1/2a, 1/2b, 1/2c, and 4b and for determination of the corresponding lineages (Table S1).

### Antibiotic susceptibility

All isolates were screened for AB^R^ phenotypes by broth microdilution testing according to the approved CLSI guidelines M45-A2 for *L. monocytogenes* (Jorgensen et al. 2010). Minimal inhibitory concentration (MIC) values values that were classified as intermediate or resistant (Table2) were tested three times. A microarray targeting >100 AB^R^ genes of gram-positive bacteria (AMR+ve-2 ArrayTubes™; Alere Technologies GmbH, Jena, Germany), based on a previously published microarray (Perreten et al. 2005), was applied for genotypic resistance profiling, followed by PCR confirmation for known AB^R^ genes (Table S1).

### In vitro conjugation experiments

Transferability of resistance determinants found in this study was analyzed on nitrocellulose membrane filters (0.45 *μ*m; Millipore AG, Zug, Switzerland) and transconjugants were isolated as published before (Bertsch et al. 2013a). Streptomycin-resistant *L*. *monocytogenes* 10403S (Bishop and Hinrichs 1987) and rifampicin-resistant *Ent. faecalis* JH2-2 (Jacob and Hobbs 1974) were used as recipients and the transfer rate was calculated as transconjugants per donor.

## Results and Discussion

### Characterization of *L. monocytogenes* isolates

The majority (almost 90%) of the 383 *L. monocytogenes* isolates belonged to serovars 1/2a and 4b (Table 1). All isolates of serovars 1/2a and 1/2c (62.4%) corresponded to lineage 2, serovars 4b and 1/2b (37.6%) to lineage 1, respectively, and lineage 3 was not detected. It could be shown that lineage 2 was more common in foodborne isolates (73.4%) and lineage 1 in human isolates (51%) which is in accordance with a recent review (Orsi et al. 2011). The main serovar among foodborne isolates was 1/2a (69.1%), comparable to a study from Italy (Acciari et al. 2011) where 76.6% of *L. monocytogenes* strains from cheeses (*n *=* *47) belonged to this serovar.

**Table 1:** Serovar distribution of 383 *Listeria monocytogenes* isolates from this study.

Serovars	Origin (%)	Food (%)	Human (%)	Environment (%)
	Total number	210	155	18
1/2a	228 (59.5)	145 (69.1)	74 (47.7)	9 (50)
1/2b	28 (7.3)	11 (5.2)	17 (11)	–
1/2c	11 (2.9)	9 (4.3)	2 (1.3)	–
4b	116 (30.3)	45 (21.4)	62 (40)	9 (50)

### Antibiotic resistance

The MIC distribution of the 16 tested antibiotics from *Listeria* isolates of species *L. monocytogenes*, *L. seeligeri*, *L. innocua*, *L. ivanovii*, *L. welshimeri*, and *L. fleischmannii* sp. nov. (Bertsch et al. 2013a) as well as MIC_90_ values (where growth of 90% of the isolates is inhibited) are presented in Table 2. As there are very few official susceptibility breakpoints available for *L. monocytogenes* (Jorgensen et al. 2010), partly inconsistent values are regularly adopted from other bacterial species like enterococci and staphylococci in many publications. In this study, we adhered strictly to only designating isolates as resistant whenever MICs of antibiotics were ≥3 twofold dilution steps higher than the majority of values and therefore clearly distinguishable. Classification as intermediate was based on a previous susceptibility study with different *Listeria* species (Troxler et al. 2000). We are, however, aware that the transition from intermediate to resistant is debatable in terms of classification, especially when no resistance genes can be detected. With our approach, all isolates were susceptible to amoxicillin, erythromycin, gentamicin, kanamycin, penicillin, rifampicin, and vancomycin. Intermediate values were obtained for ampicillin, chloramphenicol, ciprofloxacin, clindamycin, norfloxacin, ofloxacin, and streptomycin. Phenotypic resistances were detectable against clindamycin (*n *=* *4), tetracycline (*n *=* *11), and trimethoprim (*n *=* *3). In total, 16 (3.1%) revealed resistance and 92 (17.6%) intermediate susceptibility to at least one tested antimicrobial substance among the 524 *Listeria* strains. Resistance was detected in four of 50 (8%) *L. innocua*, one of 25 (4%) *L. ivanovii*, seven of 383 (1.8%) *L. monocytogenes*, and in all four strains of *L. fleischmannii* sp. nov., which seem to be one single, persisting clone (Bertsch et al. 2013a). Grouped by their origin, 4.9% of all foodborne *Listeria* spp. isolates (*n *= 266) and 1.9% of the clinical isolates (*n *= 155; all *L. monocytogenes*; Table S2) displayed AB^R^, whereas none of the 105 environmental *Listeria* spp. strains (mainly from water) was resistant. The low value for all *L. monocytogenes* (1.8%) is similar to recent studies from France, where 2% of foodborne and environmental *L. monocytogenes* displayed acquired AB^R^ (Granier et al. 2011) and about 1.3% of clinical isolates from humans, respectively (Morvan et al. 2010). The observation that AB^R^ was most prevalent in *L. innocua* (8%) agrees with a previously published study (Walsh et al. 2001), where it was concluded that this might be due to a species-dependent ability to acquire resistances to antimicrobials. Intermediate values were most frequently found in *L. seeligeri* (48.2%), *L. innocua* (36%), and *L. welshimeri* (33.3%), and to a lesser extend in *L. ivanovii* (12%) and *L. monocytogenes* (11%). Only for *L. monocytogenes* occurrence of intermediate susceptibility varied depending on the isolation source and was much more prevalent among clinical isolates (25.8%; Table S2), compared with environmental samples (5.6%) and foodborne isolates (0.5%). The high number of isolates showing intermediate susceptibility values for ciprofloxacin (*n *= 49), clindamycin (*n *=* *28), and norfloxacin (*n *=* *28) (Table 2) is not surprising, as the natural population of several *Listeria* species was described as intermediate to these antibiotics before (Troxler et al. 2000). Intermediate susceptibilities against ciprofloxacin (34%) and clindamycin (27%) were, for example, also detected in *L. monocytogenes* isolates from a poultry cooking plant in the United States (Lyon et al. 2008).

**Table 2:** Distribution of MICs among *Listeria* isolates with corresponding MIC_90_ values (for *n* > 20).

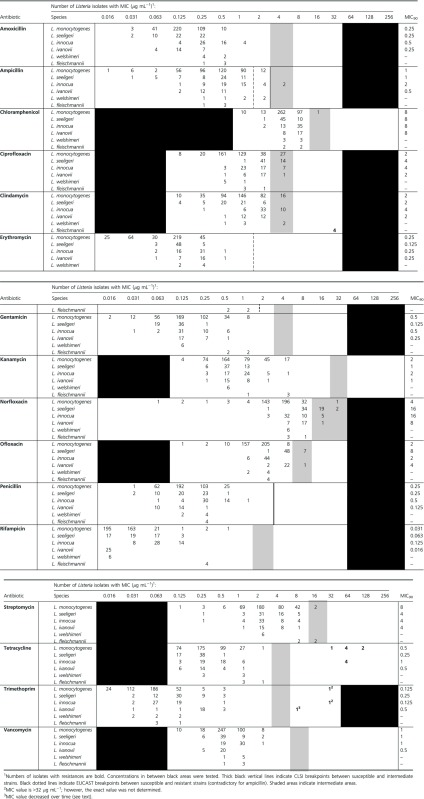

Microarray analysis (Perreten et al. 2005) and a PCR assay (Table S1) revealed that all 11 phenotypic resistances to tetracycline were encoded by the *tet*(M) gene. In eight of these isolates (three clinical and three foodborne *L. monocytogenes*, as well as two foodborne *L. innocua* strains), this ribosomal protection gene was located on a conjugative transposon of the Tn*916* family, indicated both by the presence of the transposon integrase gene *int* and by the detection of the circular transposon form by PCR (Table S1). No affiliation of the *tet*(M) gene with the Tn*916* family could be detected for one foodborne *L. monocytogenes* and two foodborne *L. innocua* strains. Phenotypic resistance to clindamycin (MIC = 32 *μ*g mL^−1^) in *L. fleischmannii* sp. nov. could not be linked to a resistance gene targeted on the microarray, as described before (Bertsch et al. 2013a). The only resistant *L. ivanovii* strain was isolated in 2008 from cheese and could be assigned to subspecies *londoniensis*. Its resistance to trimethoprim was encoded by the *dfrA* gene, which could be detected in two independent experiments both on a microarray and by PCR. However, the MIC decreased gradually from 8 to 0.25 *μ*g mL^−1^ during storage at −80°C in 33% glycerol. Thus, *dfrA* could not be detected anymore after 1 year of storage. An explanation might be that the resistance gene was encoded on a transferable element which was unstable in the isolate, a phenomenon that was observed before in *Listeria* (Hadorn et al. 1993).

With application of the EUCAST clinical breakpoints for *L. monocytogenes* from 2012 (European Committee for Antimicrobial Susceptibility Testing), 20 isolates would be classified as resistant to ampicillin due to an MIC value >1 *μ*g mL^−1^ (Table 2). Sticking to CLSI guidelines M45-A2 for *L. monocytogenes* (Jorgensen et al. 2010), where it is stated that resistance to ampicillin has not been described, we designated the two *L. innocua* strains with MICs >2 *μ*g mL^−1^ (both isolated from fish) as intermediate (Table 2). Interestingly, fish had also been the source of *L. monocytogenes* strains with MICs >2 *μ*g mL^−1^ for ampicillin in another study (Conter et al. 2009). As ampicillin is an important first-choice antibiotic for the treatment of listeriosis, we were testing these isolates for the presence of known genes encoding resistance to beta-lactam antibiotics (*bla1*, *bla2*, *blaZ*, and *mecA*) with the microarray hybridization assay (Perreten et al. 2005). However, none of these resistance genes was detected.

Notably, two isolates (0.4%) of this study were resistant to more than one antibiotic and therefore analyzed in more detail. The genetic basis of the novel transposon Tn*6198* encoding *tet*(M) and *dfrG*, which was isolated from the clinical *L. monocytogenes* isolate TTH-2007, and of the novel plasmid pDB2011 carrying *dfrD*, *erm*(A), and *spc*, detected in the foodborne *L. innocua* strain TTS-2011, was published by our group elsewhere (Bertsch et al. 2013b,c2013c). Acquired multidrug resistance seems to be exceptional in *Listeria* (Morvan et al. 2010) and was only rarely reported from France (Poyart-Salmeron et al. 1990; Quentin et al. 1990), Greece (Tsakris et al. 1997), and Switzerland (Hadorn et al. 1993). In these four cases, resistances were mediated by partly similar plasmids (>35 kb in size). In a foodborne *L. monocytogenes* isolate (designated 04CEB563LM) from France, displaying resistance to tetracycline and trimethoprim, the *tet*(M) gene seems to be encoded on a member of the Tn*916* family. However, the genetic basis of *dfrD* was not elaborated further (Granier et al. 2011).

Resistance to tetracycline due to the *tet*(M) gene was most prevalent among the isolates in this study, just as it was among *Listeria* spp. isolates in several studies before (Charpentier and Courvalin 1999). Reasons might be the extensive use of this antimicrobial worldwide, mainly in the past (Facinelli et al. 1993; Aarestrup 2012), selecting this resistance in enterococci and staphylococci, and spreading it to *Listeria* spp. by high-frequency transmissibility of *tet*(M) due to its presence on various Tn*916*-like conjugative transposons with a broad host range (Roberts and Mullany 2011). The detection of three different trimethoprim resistance genes (*dfrA*, *dfrD*, and *dfrG*) is remarkable and at the same time concerning, as there are very few reports of trimethoprim-resistant *Listeria*. This antibiotic compound is in the bactericidal drug combination trimethoprim/sulfamethoxazole (named co-trimoxazole), an option to treat listeriosis patients with intolerance to beta-lactams (Morvan et al. 2010). To the best of our knowledge, only in three *L. monocytogenes* isolates from France, a trimethoprim resistance gene (*dfrD*) could be identified so far. One of them is *L. monocytogenes* strain 04CEB563LM (see above). The *dfrD* gene was furthermore detected in the human clinical isolate U2A2348 (Morvan et al. 2010) and the environmental isolate BM4293, in which it was encoded on the 3.7 kb plasmid pIP823 (Charpentier and Courvalin 1997).

### Horizontal gene transfer potential

Transferability of all resistance genes detected in this study was tested in vitro by bacterial matings on filters. Based on MIC values obtained with a broth microdilution test, donors and transconjugants were selected by adding appropriate amounts of antibiotics into the agar medium. Recipient *L. monocytogenes* 10403S is resistant to streptomycin, but susceptible to tetracycline, trimethoprim, and clindamycin. Recipient *Ent. faecalis* JH2-2 contains a nontransferable resistance to rifampicin and shows low MICs for tetracycline, trimethoprim, and clindamycin. All donors were susceptible to streptomycin and rifampicin. Successful filter mating experiments along with transfer rates are listed in Table 3. All five *L. monocytogenes* strains harboring *tet*(M) on a transposon of the Tn*916* family transferred this transposon in similar frequencies. As expected, the strains containing *tet*(M) but lacking the *int* gene were unable to transfer the resistance gene.

**Table 3:** Combinations of donors and recipients applied in filter matings and obtained transfer rates.

*Listeria* donor species	Resistance^1^	Recipient^2^	Selection^3^	Transfer rates^4^
*L. monocytogenes*	*tet*(M) + *int*	10403S (STR^R^)	TET_26_ + STR_26_	2.5 × 10^−8^ to 5.7 × 10^−6^
		JH2-2 (RIF^R^)	TET_26_ + RIF_13_	9.4 × 10^−8^ to 1.6 × 10^−7^
*L. innocua*	*tet*(M) + *int*	10403S (STR^R^)	TET_26_ + STR_26_	2.3 × 10^−8^ to 1.1 × 10^−5^
		JH2-2 (RIF^R^)	TET_26_ + RIF_13_	5.4 × 10^−8^ to 2.4 × 10^−7^
*L. fleischmannii* sp. nov.^5^	CLI^R^	10403S (STR^R^)	CLI_13_ + STR_26_	3.7 × 10^−6^ to 5.3 × 10^−5^
		JH2-2 (RIF^R^)	CLI_13_ + RIF_13_	5.1 × 10^−7^ to 8.4 × 10^−7^

1 Resistances to tetracycline [*tet*(M)] + integrase (*int*) and clindamycin (CLI^R^) were transferred.

2 Recipient *Listeria monocytogenes* 10403S is resistant to streptomycin (STR^R^) and *Enterococcus faecalis* JH2-2 to rifampicin (RIF^R^), respectively.

3 Selection occurred on agar medium supplemented with 26 *μ*g mL^−1^ tetracycline (TET_26_), 26 *μ*g mL^−1^ streptomycin (STR_26_), 13 *μ*g mL^−1^ rifampicin (RIF_13_), and/or 13 *μ*g mL^−1^ clindamycin (CLI_13_).

4 Transfer rates as transconjugants per donor.

5 Transfer of CLI^R^ from *L. fleischmannii* sp. nov. DSM 24998^T^ was mentioned before (Bertsch et al. 2013a).

The transfer rates of *tet*(M) both from *L. monocytogenes* and *L. innocua* donors to *L. monocytogenes* 10403S and *Ent. faecalis* JH2-2 were between 10^−8^ and 10^−5^ transconjugants per donor. Frequencies were higher (up to 10^−5^) in conjugation experiments between *Listeria* spp., whereas transfer to *Ent*. *faecalis* JH2-2 occurred less frequently (10^−8^ to 10^−7^). The differences in these values depending on the recipient have also been reported before (Facinelli et al. 1993), where *tet*(M) was transferred from *L. innocua* donors to both *L. monocytogenes* and *L. innocua* recipients with frequencies of 2 × 10^−5^ and to *Ent*. *faecalis* JH2-2 with frequencies of 10^−7^, respectively. The high-level clindamycin resistance detected in all four strains of *L. fleischmannii* sp. nov. was also transferred more frequently to *Listeria* recipients (up to 10^−5^) than to *Enterococcus* recipients (10^−7^). Transfer of Tn*6198* from *L. monocytogenes* TTH-2007 to the same recipients occurred in frequencies between 10^−9^ and 10^−6^ (Bertsch et al. 2013b).

In conclusion, the relatively low number of AB^R^ in all *Listeria* (3.1%) and *L. monocytogenes* strains (1.8%) from this work is in agreement with previous studies from Europe and does not implicate an apparent increase of AB^R^ in this genus in this decade. However, next to the relatively common *tet*(M) gene, more detailed parallel studies revealed the presence of a presumably novel resistance to clindamycin (Bertsch et al. 2013a), of the novel conjugative transposon Tn*6198* (Bertsch et al. 2013b), and of the novel, broad host-range, multidrug resistance plasmid pDB2011 (Bertsch et al. 2013c) among the 524 *Listeria* strains analyzed during this study. Even though no genes conferring resistance to ampicillin were detected in this study, the elevated MIC values of 4 *μ*g mL^−1^ might warrant an increase in dosages for the successful treatment of listeriosis. The trend of this elevation was also observed in a huge study among clinical *L. monocytogenes* strains (*n *=* *4816) from France over the last decades (Morvan et al. 2010). In contrast, we did not detect alarming MIC values for the other first-choice antibiotics amoxicillin and gentamicin.

The fact that all novel resistance elements were transferable and that MICs of ampicillin are at the edge of resistance reinforces the necessity of a broad surveillance of *L. monocytogenes* to detect changes in antibiotic susceptibility as early as possible. In addition, the other species of the genus *Listeria* must not be forgotten, as they can constitute reservoirs of antibiotic resistance.

## Supporting Information

**Table S1.** PCR assays with primers used in this study. **Table S2.** Distribution of MICs among 155 clinical *Listeria monocytogenes* isolates with corresponding MIC_90_ values.
