# Supplementary material for: Antimicrobial susceptibility and antibiotic resistance gene transfer analysis of foodborne, clinical, and environmental Listeria spp. isolates including Listeria monocytogenes
Source: Microbiologyopen. 2014 Jan 2;3(1):118–27. doi: 10.1002/mbo3.155 (PMC3937734; doi:10.1002/mbo3.155)
Supplement: Supplementary file 1 — Table S1. PCR assays with primers used in this study. Table S2. Distribution of MICs among 155 clinical Listeria monocytogenes isolates with corresponding MIC90 values. [file mbo30003-0118-sd1.docx]

## Supporting Information

## Table S1 PCR assays with primers used in this study

| **Primer** | **Sequence (5’ 🡪 3’)** | **Target** | **Reference** |
| --- | --- | --- | --- |
| lmo0737-For | AGGGCTTCAAGGACTTACCC | Serovars 1/2a, 1/2c, 3a and 3c | Doumith *et al*. (2004) |
| lmo0737-Rev | ACGATTTCTGCTTGCCATTC |  |  |
| lmo1118-For | AGGGGTCTTAAATCCTGGAA | Serovars 1/2c and 3c | Doumith *et al*. (2004) |
| lmo1118-Rev | CGGCTTGTTCGGCATACTTA |  |  |
| ORF2819-For | AGCAAAATGCCAAAACTCGT | Serovars 1/2b, 3b, 4b, 4d and 4e | Doumith *et al*. (2004) |
| ORF2819-Rev | CATCACTAAAGCCTCCCATTG |  |  |
| ORF2110-For | AGTGGACAATTGATTGGTGAA | Serovars 4b, 4d and 4e | Doumith *et al*. (2004) |
| ORF2110-Rev | CATCCATCCCTTACTTTGGAC |  |  |
| *prs*-For | GCTGAAGAGATTGCGAAAGAAG | All *Listeria* species | Doumith *et al*. (2004) |
| *prs*-Rev | CAAAGAAACCTTGGATTTGCGG |  |  |
| *actA*1-f | AATAACAACAGTGAACAAAGC | Lineage 1 | Ward *et al*. (2004) |
| *actA*1-r | TATCACGTACCCATTTACC |  |  |
| *plcB*2-f | TTGTGATGAATACTTACAAAC | Lineage 2 | Ward *et al*. (2004) |
| *plcB*2-r | TTTGCTACCATGTCTTCC |  |  |
| *actA*3-f | CGGCGAACCATACAACAT | Lineage 3 | Ward *et al*. (2004) |
| *plcB*3-r | TGTGGTAATTTGCTGTCG |  |  |
| *tet*(M) forward | AGTTTTAGCTCATGTTGATG | *tet*(M) | Doherty *et al*. (2000) |
| *tet*(M) reverse | TCCGACTATTTGGACGACGG |  |  |
| *int* forward | GCGTGATTGTATCTCACT | transposon integrase *int* | Doherty *et al*. (2000) |
| *int* reverse | GACGCTCCTGTTGCTTCT |  |  |
| dfrG-F | TTTCTTTGATTGCTGCGATG | *dfrG* | Bertsch *et al*. (2013a) |
| dfrG-R | CCCTTTTTGGGCAAATACCT |  |  |
| dfrD-F | GGGCAGATTTGTTTAGTAAGG | *dfrD* | Bertsch *et al*. (2013b) |
| dfrD-R | GTATCTCCTTCGAATTCATGATG |  |  |
| dfrA-F | CCTTGGCACTTACCAAATG | *dfrA* | Perreten *et al*. (2005) |
| dfrA-R | CTGAAGATTCGACTTCCC |  |  |
| spc-F | CCAAATCAAGCGATTCAAAC | *spc* | Bertsch *et al*. (2013b) |
| spc-R | TAACGAGTGCTTTCACCT |  |  |
| ermA-F | AGTGACATTTGCATGCTTC | *erm*(A) | Bertsch *et al*. (2013b) |
| ermA-R | ATCGGATCAGGAAAAGGAC |  |  |
| L1 | CGTGAAGTATCTTCCTACAGT | Tn*916* | Manganelli *et al.* (1995) |
| R1 | GGATAAATCGTCGTATCAAAG | (G instead of C at beginning) |  |
|  |  |  |  |

**References**

Bertsch, D., Uruty, A., Anderegg, J., Lacroix, C., Perreten, V. and Meile, L. (2013a) Tn*6198*, a novel transposon containing the trimethoprim resistance gene *dfrG* embedded into a Tn*916* element in *Listeria monocytogenes*. *J Antimicrob Chemother* **68**, 986-991.

Bertsch, D., Anderegg, J., Lacroix, C., Meile, L. and Stevens, M.J.A. (2013b) pDB2011, a 7.6 kb multidrug resistance plasmid from *Listeria innocua* replicating in Gram-positive and Gram-negative hosts. *Plasmid*, first published online 14 June 2013 as doi: 10.1016/j.plasmid.2013.06.001.

Doherty, N., Trzcinski, K., Pickerill, P., Zawadzki, P. and Dowson, C.G. (2000) Genetic diversity of the *tet*(M) gene in tetracycline-resistant clonal lineages of *Streptococcus pneumoniae*. *Antimicrob Agents Chemother* **44**, 2979-2984.

Doumith, M., Buchrieser, C., Glaser, P., Jacquet, C. and Martin, P. (2004) Differentiation of the major *Listeria monocytogenes* serovars by multiplex PCR. *J Clin Microbiol* **42**, 3819-3822.

Manganelli, R., Romano, L., Ricci, S., Zazzi, M. and Pozzi, G. (1995) Dosage of Tn916 circular intermediates in *Enterococcus faecalis*. *Plasmid* **34**, 48-57.

Perreten, V., Vorlet-Fawer, L., Slickers, P., Ehricht, R., Kuhnert, P. andFrey, J. (2005) Microarray-based detection of 90 antibiotic resistance genes of gram-positive bacteria. *J Clin Microbiol* **43**, 2291-2302.

Ward, T.J., Gorski, L., Borucki, M.K., Mandrell, R.E., Hutchins, J. and Pupedis, K. (2004) Intraspecific phylogeny and lineage group identification based on the *prfA* virulence gene cluster of *Listeria monocytogenes*. *J Bacteriol* **186**, 4994-5002.

## Table S2 Distribution of MICs among 155 clinical *Listeria monocytogenes* isolates with corresponding MIC_90_-values

|  |  |  |  | **Number of isolates with MIC (µg ml^-1^)*:** | | | | | | | | |  |  |  |  |
| --- | --- | --- | --- | --- | --- | --- | --- | --- | --- | --- | --- | --- | --- | --- | --- | --- |
| **Antibiotic** | **0.016** | **0.031** | **0.063** | **0.125** | **0.25** | **0.5** | **1** | **2** | **4** | **8** | **16** | **32** | **64** | **128** | **256** | **MIC_90_** |
| **Amoxicillin** |  | 3 | 23 | 63 | 58 | 8 |  |  |  |  |  |  |  |  |  | 0.25 |
| **Ampicillin** | 1 | 5 | 1 | 10 | 32 | 55 | 44 | 7 |  |  |  |  |  |  |  | 1 |
| **Chloramphenicol** |  |  |  |  |  |  | 10 | 13 | 99 | 33 |  |  |  |  |  | 8 |
| **Ciprofloxacin** |  |  |  | 8 | 11 | 40 | 37 | 32 | 27 |  |  |  |  |  |  | 4 |
| **Clindamycin** |  |  |  | 3 | 6 | 30 | 57 | 44 | 15 |  |  |  |  |  |  | 2 |
| **Erythromycin** | 25 | 64 | 16 | 34 | 16 |  |  |  |  |  |  |  |  |  |  | 0.25 |
| **Gentamicin** | 2 | 8 | 17 | 66 | 44 | 14 | 4 |  |  |  |  |  |  |  |  | 0.5 |
| **Kanamycin** |  |  |  | 3 | 28 | 59 | 36 | 19 | 10 |  |  |  |  |  |  | 2 |
| **Norfloxacin** |  |  | 1 | 2 | 1 | 2 | 1 | 25 | 95 | 28 |  |  |  |  |  | 8 |
| **Ofloxacin** |  |  |  | 1 | 2 | 9 | 42 | 94 | 7 |  |  |  |  |  |  | 2 |
| **Penicillin** |  |  | 16 | 69 | 53 | 17 |  |  |  |  |  |  |  |  |  | 0.5 |
| **Rifampicin** | 84 | 55 | 13 |  | 2 | 1 |  |  |  |  |  |  |  |  |  | 0.063 |
| **Streptomycin** |  |  |  | 1 | 3 | 4 | 20 | 56 | 38 | 31 | 2 |  |  |  |  | 8 |
| **Tetracycline** |  |  |  | 46 | 47 | 47 | 12 |  |  |  |  |  | **2** | **1** |  | 0.5 |
| **Trimethoprim** | 24 | 44 | 60 | 21 | 3 | 2 |  |  |  |  |  | **1†** |  |  |  | 0.125 |
| **Vancomycin** |  |  |  | 9 | 17 | 84 | 37 | 8 |  |  |  |  |  |  |  | 1 |

*Numbers of isolates with resistances are bold. Concentrations in between black areas were tested. Thick black vertical lines indicate CLSI breakpoints between susceptible and intermediate strains. Black dotted lines indicate EUCAST breakpoints between susceptible and resistant strains (contradictory for ampicillin). Shaded areas indicate intermediate areas.

†MIC-value is >32 µg ml^-1^; however, the exact value was not determined.

**Reference**

Jorgensen, J.H., Hindler, J.A., Bernard, K., Citron, D.M., Cockerill, F.R., Fritsche, T.R., Funke, G., Heine, H. *et al.* (2010) Methods for antimicrobial dilution and disk susceptibility testing of infrequently isolated or fastidious bacteria. Approved guideline--2nd edition. CLSI document M45-A2, vol. 30, no. 18. Pennsylvania: Clinical and Laboratory Standards Institute.
